# Differences in Gene Expression Profile of Primary Tumors in Metastatic and Non-Metastatic Papillary Thyroid Carcinoma—Do They Exist?

**DOI:** 10.3390/ijms21134629

**Published:** 2020-06-29

**Authors:** Sylwia Szpak-Ulczok, Aleksandra Pfeifer, Dagmara Rusinek, Malgorzata Oczko-Wojciechowska, Malgorzata Kowalska, Tomasz Tyszkiewicz, Marta Cieslicka, Daria Handkiewicz-Junak, Krzysztof Fujarewicz, Dariusz Lange, Ewa Chmielik, Ewa Zembala-Nozynska, Sebastian Student, Agnieszka Kotecka-Blicharz, Aneta Kluczewska-Galka, Barbara Jarzab, Agnieszka Czarniecka, Michal Jarzab, Jolanta Krajewska

**Affiliations:** 1 Nuclear Medicine and Endocrine Oncology Department; Maria Sklodowska-Curie National Research Institute of Oncology Gliwice Branch, 44-101 Gliwice, Poland; sylwia.szpak-ulczok@io.gliwice.pl (S.S.-U.); daria.handkiewicz-junak@io.gliwice.pl (D.H.-J.); agnieszka.kotecka-blicharz@io.gliwice.pl (A.K.-B.); aneta.kluczewska-galka@io.gliwice.pl (A.K.-G.); barbara.jarzab@io.gliwice.pl (B.J.); 2 Department of Genetic and Molecular Diagnostics of Cancer, Maria Sklodowska, Curie National Research Institute of Oncology Gliwice Branch, 44-101 Gliwice, Poland; aleksandra.pfeifer@io.gliwice.pl (A.P.); dagmara.rusinek@io.gliwice.pl (D.R.); malgorzata.oczko-wojciechowska@io.gliwice.pl (M.O.-W.); malgorzata.kowalska@io.gliwice.pl (M.K.); tomasz.tyszkiewicz@io.gliwice.pl (T.T.); marta.cieslicka@io.gliwice.pl (M.C.); 3 Institute of Automatic Control, Silesian University of Technology, 44-100 Gliwice, Poland; krzysztof.fujarewicz@polsl.pl (K.F.); sebastian.student@polsl.pl (S.S.); 4 Tumor Pathology Department; Maria Sklodowska, Curie National Research Institute of Oncology Gliwice Branch, 44-101 Gliwice, Poland; dariusz.lange@io.gliwice.pl (D.L.); ewa.chmielik@io.gliwice.pl (E.C.); ewa.zembala-nozynska@io.gliwice.pl (E.Z.-N.); 5 The Oncologic and Reconstructive Surgery Clinic; Maria Sklodowska, Curie National Research Institute of Oncology Gliwice Branch, 44-101 Gliwice, Poland; agnieszka.czarniecka@io.gliwice.pl; 6 Breast Unit; Maria Sklodowska-Curie National Research Institute of Oncology Gliwice Branch, 44-101 Gliwice, Poland; michal.jarzab@io.gliwice.pl

**Keywords:** papillary thyroid cancer, distant metastases, gene expression profile, microarray

## Abstract

Molecular mechanisms of distant metastases (M1) in papillary thyroid cancer (PTC) are poorly understood. We attempted to analyze the gene expression profile in PTC primary tumors to seek the genes associated with M1 status and characterize their molecular function. One hundred and twenty-three patients, including 36 M1 cases, were subjected to transcriptome oligonucleotide microarray analyses: (set A—U133, set B—HG 1.0 ST) at transcript and gene group level (limma, gene set enrichment analysis (GSEA)). An additional independent set of 63 PTCs, including 9 M1 cases, was used to validate results by qPCR. The analysis on dataset A detected eleven transcripts showing significant differences in expression between metastatic and non-metastatic PTC. These genes were validated on microarray dataset B. The differential expression was positively confirmed for only two genes: *IGFBP3,* (most significant) and *ECM1*. However, when analyzed on an independent dataset by qPCR, the *IGFBP3* gene showed no differences in expression. Gene group analysis showed differences mainly among immune-related transcripts, indicating the potential influence of tumor immune infiltration or signal within the primary tumor. The differences in gene expression profile between metastatic and non-metastatic PTC, if they exist, are subtle and potentially detectable only in large datasets.

## 1. Introduction

Thyroid cancer incidence has increased in recent years. The estimated morbidity is 15.8 persons per 100,000 population (8.0 per 100,000 men and 23.3 per 100,000 women), which represents 3.0% of all new cancer cases diagnosed in the United States [1]. Better availability of sonography leads to the detection of small lesions, which are mostly clinically asymptomatic [2]. The most common type—papillary thyroid carcinoma (PTC)—constitutes 65%−93% of all thyroid cancer cases, depending on the analyzed population [3]. Five-year overall survival for thyroid cancer is 98.2% [1]. Despite the increased number of new cases, the number of deaths remains stable, and it is 0.5 per 100,000 population.

Differentiated thyroid cancer (DTC) is characterized by an excellent prognosis, with 10-year survival rates exceeding 90%. Nevertheless, 3–15% of DTC patients have distant metastases (M1) at presentation [4,5], whereas recurrent disease is diagnosed within decades in up to 30% of patients [6]. Two-third of relapses (66%) occur within the first ten years. Among them, 79% of cases have a locoregional disease, whereas 21% of cases manifest as distant metastases, mostly in the lungs (53–63%) and in the bones (19–20%). Numerous risk score systems have been developed to predict the course of the disease. These classifications are based on different clinical and histopathological data, including age at diagnosis, tumor diameter, cancer type and subtype, invasion outside thyroid capsule, lymph node, and distant metastases. The most common are TNM (tumor, nodes, metastasis) system, MACIS (Metastases, Age, Completeness of resection, Invasion [local], Size) score, and ATA (American Thyroid Association) Initial Risk Stratification System [7,8]. Surprisingly, none of these systems consider molecular features. The prognostic significance of *BRAF^V600E^* or *TERT* promoter mutations in PTC has been widely discussed recently [9,10,11,12]. The *BRAF^V600E^* mutation is the most common molecular alteration in PTC, being present in 36%-83% of PTC cases. Its impact on the PTC course is not so unequivocal. Numerous papers have pointed to a significant association between the *BRAF^V600E^* mutation and other poor prognostic factors, including older age, male gender, tumor size, extrathyroidal extension, lymph node or distant metastases, higher PTC stage, and the risk of recurrence or PTC-related death [13,14,15,16,17,18]. However, other studies did not confirm these findings [9,19]. *TERT* promoter mutations, reported for the first time in thyroid carcinoma in 2013 [20], are rare and occur in 7.5% of PTCs [21]. The coexistence of *TERT* promoter and *BRAF* mutation, frequently observed in PTC, is considered as being related to poorer prognosis and a more aggressive PTC course [22,23,24,25]. Noteworthily, some published data indicate that a negative impact on PTC prognosis is a consequence of *TERT* mutations solely [26] than their combination with the *BRAF* mutation. In contrast, other sources demonstrate that the effect of *TERT* mutations decreased or disappeared when these two mutations occurred separately [22,23,27]. Following these data, the assessment of the *BRAF^V600E^* mutation or *TERT* promoter mutation is currently being implemented into clinics, although it is still not a part of daily clinical practice. However, one should remember that the confirmation of the presence of *BRAF* or *TERT* mutation does not allow one to select patients with a high risk of distant metastases accurately and so far, does not influence clinical management either.

In contrast to our knowledge regarding the impact of mutations, as discussed above, the data linking gene expression profile and the risk of PTC spread are scarce and are mostly directed at the risk of nodal rather than distant metastases [28,29,30,31]. As nodal metastases do not preclude excellent prognosis, these attempts did not translate into clinically useful predictor. Previous studies to find at least one predictive gene expression marker of distant metastases in PTC failed. We believe we are justified to draw such a conclusion, as a small number or even no papers continuing these analyses were published.

Another important issue, showing huge progress in recent years, is the analysis of molecular pathways, bringing new light to the molecular pathogenesis of metastasis [32]. Such analyses were also carried out in thyroid carcinoma [33,34]. It is believed that understanding the interplay and deregulation in molecular pathways may be crucial for the development of new therapeutic strategies and finally lead to the improvement of long-term prognosis.

In this study, we verify a hypothesis that there exist gene expression markers in primary thyroid tumors which cause a predisposition to the occurrence of distant metastases (synchronously or metachronically), which could potentially serve as a valuable prognostic/predictive marker. Thus, in the analysis, we attempt to find differentiating genes to predict M1 in PTC.

## 2. Results

**Figure 1:**
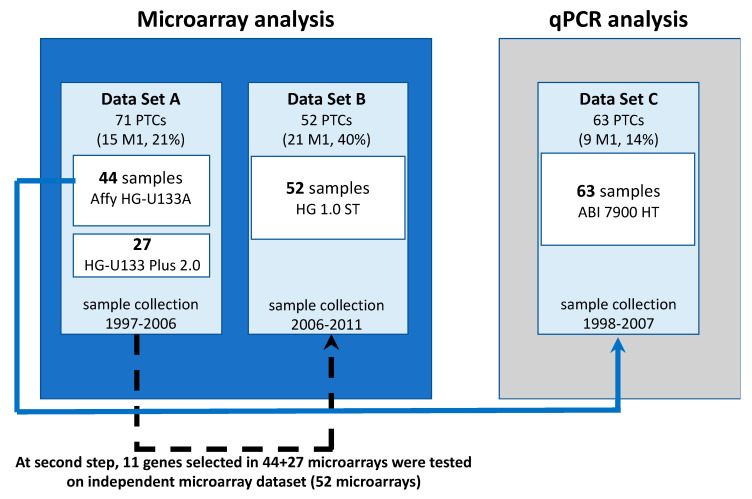
Study scheme. The set C is highlighted by a different color of the background to emphasize our resignation from this analysis during the further study course.

Our study included material, longitudinally collected intraoperatively at our center. The sample collection lasted several years, and several attempts to perform the analysis were made during the time. Simultaneously, microarray technology was developing, so the data obtained in different timepoints show a batch effect due to different reagent batches and—in part—different microarray batches and generations. Initially, the planned analysis included independent discovery and validation sets (microarrays and qPCR, respectively) (Figure 1). As the results of the initial discovery cohort analysis showed weak discrimination and validation results were inconclusive, we extended the microarray analysis to also cover the samples preliminarily used for qPCR (Dataset A). Later on, an additional dataset was collected and analyzed (Dataset B). The first set of PTC tumors was analyzed in 2005 using the HGU 133A arrays. The next set was subsequently collected and analyzed in 2009, when the previous arrays were no longer available. Thus, we used HGU133 2.0 PLUS arrays. After the second analysis, our results were still inconclusive. So, in 2014, we analyzed the third set of samples using a new generation of microarrays. The use of different platforms required careful analysis. Therefore, we decided to combine all the sets.

### 2.1. Discovery Sample Set A

The first stage of analysis was performed in microarray dataset A (71 PTC tumors) using Affymetrix Human Genome U133A Arrays (HG-U133A) or Affymetrix Human Genome U-133 Plus 2.0 Arrays (HG-U13 Plus 2.0). When differentially expressed genes were selected using Linear Models for Microarray Data (limma), 11 transcripts showed significant differences in expression (FDR adjusted *p*-value < 0.05) between PTCs with and without distant metastases (Table 1). All the genes were upregulated in metastatic PTCs.

### 2.2. Validation Sample Set B

In the next step, we performed a validation of 11 selected genes using microarray validation dataset B (52 independent PTC samples) using Affymetrix Human Gene 1.0 ST Arrays. The differential expression was positively confirmed for two genes: insulin-like growth factor binding protein 3 (*IGFBP3*) and extracellular matrix protein 1 (*ECM1*) (Bonferroni corrected p-value < 0.05). In the validation microarray dataset B, *IGFBP3* and *ECM1* have relatively large signal log ratios (SLR). The SLR of *IGFBP3* is equal to 0.83, which corresponds to a fold change (FC) equal to 1.77, and the SLR of *ECM1* is equal to 1.08, which corresponds to an FC equal to 2.11. To additionally test the significance of the result, we compared the SLR of those two genes to the SLR of other genes in the microarray—they rank in the highest percentile of differences (only 0.2% of 12,001 genes show an absolute SLR higher than *IGFBP3*, and 0.07% of genes show an absolute SLR larger than *ECM1).* Using validation microarray dataset B, we also estimated the probability that a minimum two out of 11 randomly selected genes would be positively validated, with such a high absolute SLR (absolute SLR of minimum two genes larger or equal to 0.83). The probability of such an event is lower than 0.0001, which may suggest the real association of *IGFBP3* and *ECM1* with PTC metastases.

### 2.3. Real-Time Quantitative RT-PCR Analysis (qPCR)

Albeit significant in both microarray datasets, *IGFBP3* was among the genes which were evaluated by qPCR in the first stage of our analysis (Table S1). The result was found to be negative: the median expression in M0 group was 0.0812, whereas in M1 group 0.0955 (*p* = 0.2933). As the remaining genes analyzed by qPCR were not confirmed by analysis of Gene Set A and B, and after correction for multiple comparisons, this analysis led to insignificant genes, we provide it only in Table S1.

### 2.4. Functional Gene Set Enrichment Analysis

We analyzed the differences in gene expression at the level of functional gene sets using gene set enrichment analysis (GSEA) method. In discovery Sample set A, 262 gene sets were significant (adjusted *p*-value < 0.05) within the most informative Biological Process classification, with 20−30 gene sets in remaining GO trees and KEGG database, and 11-296 gene sets in MSigDB collections (Table 2). A similar number of gene sets (260) was significant in Sample set B, and 54 of these sets were found to be significant in both dataset A and dataset B (all showed the concordant sign of normalized enrichment score, NES). As the gene sets are redundant, after eliminating the most redundant oncology terms, seven Biological Process ontology terms were found to be significantly over-represented, with NES score absolute value above 2.0 (Table 3). The top differences were seen in closely related GO terms “positive regulation of cell killing” (GO:0031343) and “natural killer cell-mediated immunity” (GO:0002228). Differences of the largest magnitude (the highest absolute NES) were seen in other immunity-related gene sets, including the positive regulation of T-cell proliferation and immunoglobulin production. They all were mirrored by a large-scale difference in the general GO term “lymphocyte-mediated immunity” (GO:0002449), with the NES score of this subgroup being −2.15. All immunity-related subsets showed negative NES scores, indicating a higher expression of immunity-related transcripts in patients without distant metastases (M0).

In contrast, only a limited number of gene sets showed a concordant large magnitude change in patients presenting with distant metastases (M1), when Sample sets A and B were compared. Only one gene set was significant both in Sample set A and B, with an NES score above 2.0 and no redundancy: “regulation of cellular amine metabolic process” (GO:0033238). This gene set is closely associated with two other potentially relevant biologically gene sets significant in Sample set A (“establishment of planar polarity”, GO:0001736, NES 1.76 and “non-canonical Wnt signaling pathway, GO:0035567, NES 1.65). However, both of these were non-significant in Sample set B analysis.

## 3. Discussion

In our study, we undertook a step-by-step analysis to find differences between M0 and M1 papillary thyroid carcinomas. As several platforms were used, with significant heterogeneity within the data obtained, at this stage, we did not decide to perform formal meta-analysis (technical aspects discussed later on). We did so also to deliberately reveal the process of the collection of data and step approach and avoid potential bias in data analysis related to our several previous attempts.

Multigene expression signatures were characterized for different neoplasms, aiming to derive clinically meaningful classifiers. However, this has been successfully achieved only in selected malignancies. Breast cancer is the most prominent example: multigene signatures show a prognostic significance. They are partially used as a predictive marker to select the patients who benefit from adjuvant chemotherapy [35]. The prognostic aspect of breast cancer survival analysis was, in the majority of datasets, based on the prediction of distant metastasis—since the very beginning, this approach has been highly successful [36,37,38]. Based on these findings, we believed a similar multigene expression signature, discriminating patients at high risk of distant metastases from low-risk individuals, could be derived from thyroid cancer primary tumors. Thereby, we decided to collect prospectively the postoperative material of patients operated at our hospital. As our institute is a tertiary reference center for thyroid carcinoma, the number of patients with metastatic disease is relatively high. In the current study, we analyzed more than a hundred patients, including 35% of patients with metastatic disease. Historically, the first group of 15 PTC patients who developed distant metastases was compared to 56 M0 patients (dataset A). The identified differentially expressed genes were validated on a group of 21 M1 and 31 M0 patients (dataset B), collected later. Importantly, within the group of metastatic PTCs, there were both patients with distant metastases at initial staging and individuals who developed late metastases in the course of the follow-up.

Our first analyses did not point to any essential differences between non-metastatic and metastatic PTCs. Initially, we considered a too short time of follow-up in a group of non-metastatic patients a major obstacle for successful analysis (considering missing cases with metastases developing further in the course of the disease). Having a relatively low number of specimens, we decided to wait for a longer follow-up time and collect further samples. Our analysis involved nine additional patients in whom metastases were diagnosed later than one year after primary treatment (up to 60 months after). Finally, in our opinion, the follow-up was long enough to select M0 patients correctly. However, almost doubling the population and lengthening of the follow-up did not result in a spectacular increase in the magnitude of observed differences.

Due to the long time necessary, on the one hand, to collect a sufficient number of metastatic PTC patients, and, on the other hand, to correctly classify M0 cases, we were forced to apply different generations of microarrays during the study. Data were analyzed using linear models for microarray data (limma), a method developed and well-tailored to multiple comparisons scenarios in a genomic setting, with the batch of microarrays included as a variable in a model. However, we observed a kind of double failure—first, only 11 genes were deemed significant (including *IGFBP3* and *ECM1),* a relatively low number to underline a biologically sound difference; second, one of the genes was earlier validated by an independent method on an independent dataset and was not significant (qPCR on an independent validation set C of PTC samples). We decided to abandon a plan of validating other genes by qPCR and carried out microarray profiling, which led to the confirmation of only two mentioned transcripts. At the moment, we consider these results as negative and not confirming our hypothesis of differences between M1 and M0 PTCs. We want to emphasize that our qPCR group (set C) reflected a more real frequency of distant metastases in PTC (it involved nine M1 patients among 63 cases analyzed (14%) whereas, in initial discovery datasets, we accumulated a larger number of M1 patients. Thus, a lack of differences in the *IGFBP3* gene by qPCR could be impacted by the sample size but provides clear information that the magnitude of change in this single marker gene would not be sufficient in routine clinical practice. Nevertheless, in our opinion, this gene is potentially important and deserves further evaluation, as the role of the IGF system in thyroid cancer has been discussed for a long time [39,40]. Regarding the *ECM1* gene, it was reported not only in thyroid cancer but also in other solid tumors. Kebebew et al. analyzed its diagnostic value in thyroid carcinoma, pointed out also for potential association with disease extent [41].

Regarding the data presented above, our initial hypothesis did not seem to be justifiable. We were able to find a subtle difference between non-metastatic and metastatic PTC only, and we were unable to confirm them by independent methods. We did not find a characteristic gene expression profile, typical for metastatic PTC, although our group analyzed by microarray the largest number of metastatic PTCs (36 cases), as already published. The major potential reason for this is the low frequency of PTC metastatic spread in the general population. It seems evident we should discuss essential data provided by The Cancer Genome Atlas (TCGA) study. This study included 496 PTC samples, among them eight metastatic cases [42]. Thus, the number of analyzed metastatic PTCs was very low (1.6%), providing insight into a relatively indolent tumor population. We believe our data may constitute an addendum to this analysis. We are able to carry out a meta-analysis of our three genomic datasets and TCGA data [12]. We are intensively seeking a validation dataset for such an analysis; we are very open for a collaborative approach (contact: malgorzata.oczko-wojciechowska@io.gliwice.pl). However, planning a study would require at least a doubling of the number of metastatic patients to derive adequate power and validation ability. In the larger dataset, a more sophisticated bioinformatic approach, including machine learning, is necessary to provide adequate multigene discrimination. Our previous experience with thyroid cancer data [43,44] showed the feasibility of this approach when applied to differences with normal thyroid tissue or other cancer histotypes. We endorse further approaches to characterize poor prognosis in thyroid cancer, as it is of utmost importance in a clinical setting. Still, we warn against commencing the study without at least hundreds of samples from metastatic patients.

A limitation that could influence our data is also the transcriptomic platform we used. Since the early commencement of our study, oligonucleotide microarrays and the algorithm used by us did not detect, for example, long non-coding RNA (lncRNA). In addition, next-generation RNA sequencing (RNA-Seq) might introduce newer transcripts. Noteworthily, TCGA-based analyses paid our attention to lncRNA, playing an essential role in PTC [45,46,47,48,49] and other malignancies [50]. Numerous papers raised the role of lncRNA in the aggressiveness/invasiveness of thyroid carcinoma [45,51,52,53,54,55]. More data regarding this issue are necessary. We did not apply this approach as some of the metastatic PTCs were fully used up and were not left for any additional transcriptomic experiments; they exist only as a U133 microarray readout. Nevertheless, one should emphasize that the number of published studies based on RNA-seq, carried out in thyroid cancer, is small. The first one, reported in 2013, included only 20 PTCs, among them no one with distant metastases [56]. Another one was the TCGA study, as described above [42]. Thus, to our best knowledge, our study includes the largest number of metastatic PTCs studied by the genomic approach. We believe we have the right to claim that the difference in the gene expression profile of PTC primary tumors between metastatic and non-metastatic patients, if any exists, is small and requires a systematic and multi-center approach. Although the number of metastatic PTC samples in our study was relatively high (45) compared to the published data, we believe that dataset size could limit the power of conclusions.

We demonstrated that the signature of high-risk metastatic PTC was not as obvious as we initially believed. It is necessary to inform other groups involved in the research of metastatic thyroid cancer that a putative further study seeking a difference between metastatic and non-metastatic PTC shall involve a larger population and a broad portfolio of molecular methods rather than the raw transcriptomic assessment. Our study was carried out in three subsequent steps. In each step, we extended the population size, and each lengthened the patients’ follow-up. However, it did not lead to conclusive findings. The issue of the publication of negative results is widely raised to limit unnecessary repetitive small size experiments and to promote cooperation between researchers and the meta-analytic approach. We are extensively searching for a partner to carry out a more extensive analysis of PTC transcriptome to provide such conclusions in the future.

The critical issue regarding the occurrence of metastases is related to the host response. It is well known that PTC metastasizes mainly to niches localized in lymph nodes, lungs, and bones. The data characterizing the features of metastatic niches (receptive to colonization by circulating tumor cells (CTC)) are growing [57]. It is still not known whether this readiness of host cells regarding metastatic colonization is related to any molecular mechanisms. Moreover, one should notice that studies on metastatic niches concern the most common cancers [58], and they did not result in clinically relevant classifiers. So far, we do not have any data regarding thyroid carcinoma. Regarding the host response, we should also consider an anti-tumor immune response with the presence of immune cells in the specimen. Unfortunately, we do not have complete data regarding tumor immune infiltration or the presence of autoimmune thyroiditis in our material. However, we would like to stress that the requirement of a high percentage of PTC cells confined the number of infiltrated stroma in the analyzed material.

The important question is whether PTCs metastasize due to any mechanism clearly distinguishable in primary tumor gross specimen. We assumed that the invasiveness of the primary tumor regarding the development of distant metastases influences the PTC course, while current data also indicate other mechanisms, including the ability of cancer cells to survive, extravasation, or colonization by CTC [59,60]. The CTCs may be a result of a very small primary tumor subclone, indistinguishable in the gross transcriptomic analysis. Although microdissection studies are feasible and were also performed by our group [61], no study has been performed using this approach in the clinical context. A high-quality RNASeq experiment may indicate the subclonal tumor structure. We are currently collecting tumors for such a study. In previous attempts, we succeeded in deriving novel markers for follicular thyroid tumors, in samples analyzed previously by microarray and qPCR [62,63]. It also cannot be excluded that metastatic spread in certain tumors is a stochastic event, or the changes are late and discriminable only when distant metastasis tissue is accessible [64]. In any of these scenarios, we cannot abandon the approaches to search for molecular predictors, as the clinically known PTC features are not sufficient to predict patient outcome in full [7]. It is also important when de-escalation of surgical treatment is contemplated or when adjuvant therapy has to be administered.

As the transcript-oriented analysis did not bring reliable results, we also approached the same problem at a gene set level. The hypothesis supporting this analysis is that coordinated changes in gene expression in certain functional gene groups could be detected with a lower number of false negatives. We applied a well-established algorithm of the gene set enrichment analysis. This analysis was carried out in Sample set A. The results were validated in Sample set B. We found at least six highly over-expressed gene sets significant in both datasets, with the expression increased in non-metastatic tumors. However, the vast majority of these genes were clearly associated with immune response. Although one can speculate that some changes come inherently from PTC cells, a more probable explanation is that they are related to lymphocyte infiltration of the primary tumor. The presence of infiltrating lymphocytes in tumor or stroma was recognized earlier in PTC and could be associated with a good prognosis [65]. However, according to many authors, these conclusions seemed controversial [66,67]. In our transcriptome-wide analysis of the PTC gene expression, we have already found a significant proportion of immune-related genes with high variability [43]. Nevertheless, it seems evident that bulk tumor genomic analyses are not an optimal method to evaluate the role of the immune response in the tumor microenvironment. As described above, we carried out a study of microdissected PTC cells and stroma. Thus, in the future, we plan to validate our results also in an independent dataset. The issue of the prognostic relevance of lymphocytic infiltration in PTC requires further studies, particularly in the era of immunotherapy in oncology [68]. We previously found in the analysis of ovarian cancer [69] that genomic studies shall be carried out in either histologically homogenous populations or consider multiparametric covariate analysis covering variability in tumor histology. Obviously, potential immune infiltration in the primary tumor is not limited to lymphocytes. One should stress the role of tumor-associated macrophages, one of the factors potentially related to the high expression of metalloproteases [70].

To conclude, the differences in gene expression profile between metastatic and non-metastatic PTC primary tumors, if they exist, are subtle and require studies involving hundreds of tumors. Potential further studies shall take into consideration confounding factors, including the immune infiltration of primary tumors.

## 4. Materials and Methods

### 4.1. Material

The whole group comprised of 186 PTC patients, among them 141 M0 (without distant metastases) and 45 M1 (with distant metastases) cases (Table 4). All patients underwent total thyroidectomy, and all but two patients underwent radioiodine therapy. One hundred and twenty-three patients were subjected to microarray analyses using different platforms: 71 patients were subjected to HG-U133A and HG-U133 Plus 2.0 (set A), whereas 52 patients were subjected to Human Gene 1.0 ST Array (set B). Set A involved 14 out of the 16 PTC tumors analyzed in our previous paper [43]. As analyzed previously [71], the percentage of cancer cells in the analyzed specimens was higher than 50%. Sixty-three patients constituted an independent validation set C, analyzed using qPCR. The details are presented in Table 4.

Microarray analysis involved a group of 123 PTC patients, among them 36 cases with distant metastases. Detailed characteristics of the study group is given in Table 5.

Due to essential technical differences regarding the microarray platforms used, the analysis was divided into two parts. Set A included data obtained by the analyzes carried out on the following types of microarrays—HG-U133A and HG-U133 Plus 2.0—whereas validation set B involved data obtained using Human Gene 1.0 ST microarrays.

The dataset A was comprised of 71 PTC patients (Table 6) at the median age of 33 years (range: 5–76 years). The median follow-up was 160 months (range: 3.6–250.8). The majority of patients were women—55 cases (77.5%). Nearly 2/3 of patients (64.8%) were diagnosed with a classic variant of PTC. T1 and T2 features were present in 57.7% and 14.1% of patients, respectively. Lymph node metastases in the central neck compartment were observed in 24 cases (43.6%), whereas metastases in the lateral neck compartment or the upper mediastinum were observed in 29 cases (40.8%). Distant metastases developed during the course of the disease in 15 patients (21.1%) (Table 7). The diagnosis of metastases was usually stated nearly five months following the primary PTC diagnosis, range from 0 (distant metastases present at diagnosis) to 60 months, based on a post-therapeutic whole-body scan, X-ray and/or CT scan, biopsy, or histopathological examination. Almost all M1 patients had lung metastases (14 out of 15 cases), 3/15 had bone metastases (vertebra or other bones), and one patient (1/15) had liver metastases. Ten out of fifteen metastases demonstrated radioiodine avidity. Complete remission (excellent treatment response according to the ATA criteria) was achieved in 1/3 patients, whereas in the remaining cases, ATA incomplete structural or indeterminate responses were confirmed. Six patients died due to thyroid cancer.

The validation dataset B included 52 PTC cases (Table 6), mainly women (61.5%), with a median age at PTC diagnosis of 47.5 years (range 17–86), with the median follow-up of 103.8 months (range 0–156.0). Classic PTC was a predominant variant. T1 and T2 features were diagnosed in 46.2% and 28.8% of cases, respectively. Lymph node metastases in the central neck compartment were present in 29 cases (56.9%), whereas they were present in the lateral neck compartment or the upper mediastinum in 26 cases (50.0%). Distant metastases occurred in 40% of cases (21 patients), among them lung metastases (20 patients), bone, central nervous system, thymus (2 persons in each localization), and liver (1 patient). More than half of the metastases (57.1%) showed radioiodine uptake. The excellent treatment response (according to ATA guidelines [7]) was obtained in 42.9% of cases, whereas in the remaining cases, the incomplete structural response was the final treatment outcome. Seven patients died, six persons due to metastatic disease, and one person because of advanced locoregional PTC.

The validation of the *IGFBP3* gene was performed on an independent PTC set, including 63 cases, among them 9 with metastatic disease (Table S2).

The use of human tissue was approved by the Bioethics Committee at Maria Sklodowska-Curie National Research Institute of Oncology Gliwice Branch. Written informed consent to analyze the tissue was obtained from all patients. All clinical data were anonymized and de-identified before the analysis.

### 4.2. Microarray Analysis

Data from three types of oligonucleotide array were selected for this study: HG-U133A, HG-U133 Plus 2.0, and Human Gene 1.0 ST Array (Affymetrix, Santa Clara, CA, United States). RNA was isolated from fresh frozen tumor fragments with the RNeasy Mini Kit (Qiagen, GmbH, Hilden, Germany) as recommended by the manufacturer. Microarray analysis was performed according to the manufacturer recommendations, different for each array type. However, the main steps of the laboratory protocol were common. In brief, RNA was the template for double-stranded cDNA synthesis, followed by transcription combined with cRNA biotinylation, cRNA fragmentation, and cRNA hybridization to the arrays. After washing (Fluidic Station 450, Affymetrix) and staining with streptavidin-phycoerythrin, conjugate arrays were scanned in the GeneChip 3000G scanner (Affymetrix).

The main difference between HG-U133 microarrays and Human Gene 1.0 arrays concerns the number of target-oriented probes. The HG-U133 type arrays contain 11 probes per transcript, which are located mainly around the 3′ end. Meanwhile, in the Human Gene 1.0 arrays, there are more probes designed to be distributed across the transcribed regions of each gene. Moreover, the HG-U133 arrays contain a perfect match probe (PM) and a mismatch probe (MM), used together to measure the abundance of mRNA transcripts. The MM probes have a 13th base that does not match the target sequence, allowing for detection of cross-hybridization. The Human Gene 1.0 arrays, in turn, represent a perfect match-only array design. Mentioned differences force the use of different algorithms in data analysis.

### 4.3. qPCR Analysis

RNA for qPCR analysis was isolated from fragments of fresh frozen PTC tumors using the RNeasy Mini Kit (Qiagen), as recommended by the manufacturer. qPCR was carried out for 28 genes (listed in Table S1) with the 7900HT Fast Real-Time PCR System (Life Technologies, Carlsbad, CA, USA) and the use of Roche Universal Probe Library (Roche, Basel, Switzerland). Primer sequences are given in Table S3 (supplementary materials). Each sample was examined in duplicates. For the normalization of qPCR data, the Pfaffl method and GeNorm application were used. Three normalization genes were selected: *EIF3S10* (eukaryotic translation initiation factor 3, subunit 10 theta), *HADHA* (hydroxyacyl-CoA dehydrogenase trifunctional multienzyme complex subunit alpha), and *UBE2D2* (ubiquitin conjugating enzyme E2 D2). The differences between M0 and M1 patients were tested with the two-tailed Mann–Whitney U test.

### 4.4. Statistical Analysis of Clinical Data

Categorical data were summarized with numbers and percentages. Continuous data were summarized with medians and ranges. Comparisons of categorical variables were performed using Fisher’s exact test. Comparisons of continuous variables were performed using the two-tailed Mann–Whitney U test. *p* values < 0.05 were considered statistically significant. Statistical analyses were performed using the R software version 3.6.2 and “tableone” package version 0.11.1. [73,74].

### 4.5. Microarray Data Analysis

We obtained three PTC microarray datasets: 44 HG-U133A microarrays, 27 HG-U133 Plus 2.0 microarrays, and 52 Human Gene 1.0 ST microarrays (123 samples in total).

Background correction, normalization and probe set summarization were done using the Robust Multichip Average (RMA) algorithm with library oligo v 1.50.0 from R v3.6.2 environment, and custom CDF files from BrainArray (ENTREZG; v24), for each of three microarray datasets separately [75,76,77]. Twelve thousand and one genes, common for all three types of microarray, were selected and used in further analysis.

To create microarray dataset A, we combined HG-133A and HG-U133 Plus 2.0 microarray datasets and removed a batch effect associated with microarray type using linear models for microarray data (limma) from limma 3.42.2 library [78]. We selected differentially expressed genes, using limma, with a batch factor incorporated in the linear model. *p*-values were adjusted for multiple testing by the Benjamini and Hochberg false discovery rate (FDR) method [79]. Corrected *p*-values < 0.05 were considered statistically significant.

The validation microarray dataset B was comprised of Human Gene 1.0 ST microarrays. We performed differential gene analysis using limma. *p*-values were adjusted using Bonferroni correction, on a subset of 11 preselected genes. Corrected *p*-values < 0.05 were considered statistically significant. Using the validation microarray dataset, we also estimated the probability that from 11 randomly selected genes, a minimum of two would be positively validated, with absolute SLR larger than or equal to 0.83. We performed 10,000 iterations. In each iteration, we randomly selected 11 genes (out of 12,001 genes analyzed on validation microarray dataset B), obtained *p*-values from differential gene analysis, and applied Bonferroni correction. Furthermore, in each iteration, we checked whether a minimum of two genes were significant (according to criterion: Adjusted *p*-value < 0.05) and whether a minimum of two of them showed an SLR above 0.83. We calculated the probability as the number of iterations that fulfilled the criteria divided by the number of all iterations.

### 4.6. Gene Set Enrichment Analysis

Gene set enrichment analysis (GSEA) was performed using Gene Ontology (GO) Biological Process (BP), GO Molecular Function (MF), GO Cellular Compartment (CC), the Kyoto Encyclopedia of Genes and Genomes (KEGG), and two collections from Molecular Signatures Database v7.1 (MSigDB) [80,81]: collection “hallmark gene sets” (H) and sub-collection “chemical and genetic perturbations” (CGP) being a part of the collection “curated gene sets” (C2) [82,83,84].

The analysis was performed in the R environment, using clusterProfiler 3.14.3 library [85]. The t-statistic obtained in limma analysis was used as a gene ranking metric. The *p*-values were calculated based on 10,000 random gene set permutations. Gene sets that involved between 10 and 600 genes were analyzed. *p*-values were adjusted for multiple testing by the Benjamini and Hochberg false discovery rate (FDR) method. Gene sets with corrected *p*-values < 0.05 were considered statistically significant. Redundancy amongst GO terms was removed using the ClusterProfiler simplify function with the similarity measure cut-off of 7.0, and the *p*-value used as the deciding variable. To test whether there is a significant overlap between significant gene sets obtained in datasets A and B, the Fisher exact test was used.

## Figures and Tables

**Table 1:** Genes differentially expressed between papillary thyroid cancer (PTC) with and without distant metastases. Genes were selected on microarray dataset A (71 PTC samples) and validated on microarray validation dataset B (52 PTC samples).

			Microarray Dataset A (*n* = 71)					Microarray Validation Dataset B (*n* = 52)		
Gene Symbol	Entrez Gene ID	Gene Name	*p*-Value	FDR-Adjusted *p*-Value	Log2-Tranformed Mean Expression in Metastatic PTC	Log2-Tranformed Mean Expression in Non-Metastatic PTC	Fold Change (Metastatic vs. Non-Metastatic)	*p*-Value	Bonferroni-Adjusted *p*-Value	Fold Change (Metastatic vs. Non-Metastatic)
*IGFBP3*	3486	insulin like growth factor binding protein 3	1.59 × 10^−6^	0.010	9.2	7.9	2.6	1.00 × 10^−4^	0.001	1.8
*ECM1*	1893	extracellular matrix protein 1	2.17 × 10^−5^	0.029	10.5	8.3	4.5	4.30 × 10^−3^	0.047	2.1
*SLC26A3*	1811	solute carrier family 26 member 3	2.67 × 10^−5^	0.032	3.3	2.9	1.3	2.57 × 10^−2^	0.283	1.1
*YAF2*	10138	YY1 associated factor 2	2.60 × 10^−6^	0.010	5.5	5.0	1.4	3.25 × 10^−2^	0.358	1.2
*CHST7*	56548	carbohydrate sulfotransferase 7	2.98 × 10^−5^	0.033	6.5	5.7	1.7	3.30 × 10^−2^	0.363	1.2
*TMEM255A*	55026	transmembrane protein 255A	9.09 × 10^-6^	0.016	5.2	3.7	2.8	3.98 × 10^−2^	0.437	1.5
*MAP2K1*	5604	mitogen-activated protein kinase kinase 1	1.09 × 10^−5^	0.016	9.4	8.8	1.5	6.12 × 10^−2^	0.673	1.1
*PLOD1*	5351	procollagen-lysine,2-oxoglutarate 5-dioxygenase 1	5.73 × 10^−6^	0.016	7.8	7.3	1.4	1.20 × 10^−1^	1.000	1.1
*SMYD3*	64754	SET and MYND domain containing 3	7.46 × 10^−6^	0.016	8.5	7.8	1.6	3.94 × 10^−1^	1.000	1.1
*STX6*	10228	syntaxin 6	9.24 × 10^−6^	0.016	6.6	6.3	1.2	8.58 × 10^−1^	1.000	1.0
*RNPEP*	6051	arginyl aminopeptidase	8.92 × 10^−7^	0.010	8.9	8.5	1.3	8.73 × 10^−1^	1.000	1.0

**Table 2:** Comparison of significant gene sets in gene set enrichment analysis (GSEA) of Sample set A and Sample set B.

Gene Set	Number of Significant Gene Groups			Association between Lists of Significant Gene Sets;*p*-Value (Exact Fisher Test)
	Sample Set A	Sample Set B	Significant in Both Sets, Showing a Concordant NES	
GO Biological Process	262	260	54	<2.2 × 10^−^^16^
GO Cellular Component	27	78	5	0.2034
GO Molecular Function	30	115	5	0.03644
KEGG Pathways	20	31	5	0.007402
MSigDB:H	11	16	5	0.2972
MSigDB:C2:CPG	296	267	103	<2.2 × 10^−^^16^

**Table 3:** Selected significant Gene Ontology Biological Process gene sets. Only gene sets significant in both Sample set A and validation Set B were listed and limited to gene sets with absolute NES above 2.0 in Sample Set A, after the exclusion of redundant sets.

GO Biological Process Gene Sets ID and Description (Set Size)	Sample Set A		Sample Set B	
	NES	adj. *p* Value	NES	adj. *p* Value
GO:0031343 positive regulation of cell killing (57)	−2.38	0.0175	−1.74	0.0297
GO:0002228 natural killer cell mediated immunity (49)	−2.35	0.0175	−1.87	0.0163
GO:0046641 positive regulation of alpha-beta T cell proliferation (18)	−2.32	0.0175	−1.84	0.0495
GO:0002377 immunoglobulin production (94)	−2.17	0.0175	−2.07	0.0163
GO:0002449 lymphocyte mediated immunity (214)	−2.15	0.0175	−1.68	0.0163
GO:0050853 B cell receptor signaling pathway (52)	−2.06	0.0175	−2.18	0.0163
GO:0033238 regulation of cellular amine metabolic process (72)	2.02	0.0175	1.98	0.0163

**Table 4:** Clinical characteristic of the whole PTC group.

	All PTC Patients	M1 Patients	M0 Patients	*p*-Value
Data set: All	186	45	141	
A	71	15	56	
B	52	21	31	
C	63	9	54	
Median age at diagnosis (years) (range)	43 (5–86)	42 (5–81)	43 (10–86)	0.925
Median follow-up (months) (range)	140.0 (2.1–250.9)	94.8 (3.2–250.9)	146.2 (2.1–220.0)	<0.001
Sex				0.012
male	50 (26.9%)	19 (42.2%)	31 (22.0%)	
female	136 (73.1%)	26 (57.8%)	110 (78.0%)	
PTC histological subtype				0.301
classic	141 (75.8%)	35 (77.8%)	106 (75.2%)	
follicular	37 (19.9%)	10 (22.2%)	27 (19.1%)	
other	8 (4.3%)	0 (0.0%)	8 (5.7%)	
T feature ^1^ (primary tumor)				<0.001
T1	104 (55.9%)	11 (24.4%)	93 (66.0%)	
T2	36 (19.4%)	9 (20.0%)	27 (19.1%)	
T3	20 (10.8%)	11 (24.4%)	9 (6.4%)	
T4	18 (9.7%)	10 (22.2%)	8 (5.7%)	
Tx	8 (4.3%)	4 (8.9%)	4 (2.8%)	
Lymph node metastases				
central neck compartment and upper mediastinum (N1a)	77 (44.4%)	33 (73.3%)	44 (31.2%)	<0.001
lateral neck compartment and retropharyngeal lymph nodes (N1b)	76 (40.9%)	35 (77.8%)	41 (29.1%)	<0.001
Recurrence after primary treatment	14 (7.5%)	3 (6.7%)	11 (7.8%)	>0.999
PTC- related death	16 (8.6%)	15 (33.3%)	1 (0.7%)	<0.001
Postoperative risk stratification ^2^				<0.001
very low risk	12 (6.5%)	0 (0.0%)	12 (8.5%)	
low risk	63 (33.9%)	0 (0.0%)	63 (44.7%)	
intermediate risk	0 (0.0%)	0 (0.0%)	0 (0.0%)	
high risk	111 (59.7%)	45 (100%)	66 (46.8%)	

^1^ The patients were staged according to the 8th UICC/AJCC TNM Edition (2016). ^2^ Postoperative stratification according to the ETA consensus 2006 [72]. PTC—papillary thyroid cancer; M1—distant metastases present; M0—distant metastases absent.

**Table 5:** Clinical characteristic of the whole microarray group.

	All PTC Patients	M1 Patients	M0 Patients	*p*-Value
Patients number	123	36	87	
Median age at diagnosis (years) (range)	40.0 (5–86)	43.5 (5–81)	38.0 (10–86)	0.284
Median follow-up (months) (range)	127 (0.0–250.8)	93.0 (3.6–250.8)	144.0 (0.0–219.6)	<0.001
Sex:				0.191
male	36 (29.3%)	14 (38.9%)	22 (25.3%)	
female	87 (70.7%)	22 (61.1%)	65 (74.7%)	
PTC histological subtype				0.271
classic	90 (73.2%)	29 (80.6)	61 (70.1%)	
other	33 (26.8%)	7 (19.4%)	26 (29.9%)	
T feature (primary tumor):				<0.001
T1	65 (52.8%)	10 (27.8%)	55 (63.2%)	
T2	25 (20.3%)	7 (19.4%)	18 (20.7%)	
T3	12 (9.8%)	9 (25.0%)	3 (3.4%)	
T4	15 (12.2%)	8 (22.2%)	7 (8.0%)	
Tx	6 (4.9%)	2 (5.6%)	4 (4.6%)	
Lymph node metastases:				
central neck compartment and upper mediastinum (N1a)	53 (50.0%)	26 (83.9%)	27 (36.0%)	<0.001
lateral neck compartment and retropharyngeal (N1b)	55 (44.7%)	28 (77.8%)	27 (31.0%)	<0.001
Recurrence after primary treatment	8 (6.5%)	2 (5.6%)	6 (6.9%)	>0.999
PTC- related death	13 (10.6%)	12 (33.3%)	1 (1.1%)	<0.001

The patients were staged according to the 8th UICC/AJCC TNM Edition (2016). PTC—papillary thyroid cancer; M1—distant metastases present; M0—distant metastases absent.

**Table 6:** Clinical characteristic of the whole group with considering microarray platform used.

	Data A	Data B	*p*-Value
	HG-U133A + HG-U133 Plus 2.0	Human Gene 1.0 ST Array	
Patients number	71	52	
Median age at PTC diagnosis (years) (range)	33.0 (5.0–76.0)	47.5 (17.0–86.0)	0.004
Median follow-up (months) (range)	160.8 (3.6–250.8)	103.8 (0—156.0)	<0.001
Sex:			0.071
male	16 (22.5%)	20 (38.5%)	
female	55 (77.5%)	32 (61.5%)	
PTC histological subtype			0.023
classic	46 (64.8%)	44 (84.6%)	
other	25 (35.2%)	8 (15.4%)	
T feature (primary tumor):			0.042
T1	41 (57.7%)	24 (46.2%)	
T2	10 (14.1%)	15 (28.8%)	
T3	5 (7.0%)	7 (13.5%)	
T4	9 (12.7%)	6 (11.5%)	
Tx	6 (8.5%)	0 (0.0%)	
Lymph node metastases:			
central neck compartment and upper mediastinum (N1a)	24 (43.6%)	29 (56.9%)	0.243
lateral neck compartment and retropharyngeal (N1b)	29 (40.8%)	26 (50.0%)	0.361
Recurrence after primary treatment	3 (4.2%)	5 (9.6%)	0.281
Distant metastases	15 (21.1%)	21 (40.4%)	0.034
PTC-related death	6 (8.5%)	7 (13.5%)	0.390

The patients were staged according to the 8th UICC/AJCC TNM Edition (2016). PTC—papillary thyroid cancer.

**Table 7:** Characteristics of M1 PTC patients analyzed by microarrays.

	All Microarray Platforms Together	Data A	Data B	*p*-Value
		HG-U133A + HG-U133 Plus 2.0	Human Gene 1.0 ST Array	
Patients number	36	15	21	
Median follow-up (months) (range)	93.0 (3.6–250.8)	87.6 (3.6–250.8)	94.8(4.8–156.0)	0.923
Median time to M1 diagnosis (months) (range)	4.8 (0.0–60.0)	4.8 (0.0–60.0)	4.8(0.0–39.6)	0.832
Localization of metastases:				
Lungs	34 (94.4%)	14 (93.3%)c	20.0 (95.2%)	>0.999
Bones	4 (11.1%)	2 (13.3%)	2 (9.5%)	>0.999
CNS	2 (5.6%)	0 (0%)	2 (9.5%)	0.500
Liver	2 (5.6%)	1 (6.7%)	1 (4.8%)	>0.999
Thymus	2 (5.6%)	0 (0%)	2 (9.5%)	>0.999
RAI avidity				0.330
positive	21 (58.3%)	9 (60.0%)	12 (57.1%)	
negative	8 (22.2%)	5 (33.3%)	3 (14.3%)	
loss of RAI uptake at PTC progression	5 (13.8%)	1 (6.7%)	4 (19.0%)	
no data	2 (5.6%)	0 (0.0%)	2 (9.5%)	
ATA treatment response^*^				0.152
excellent	14 (38.9%)	5 (33.3%)	9 (42.9%)	
incomplete biochemical	0 (0%)	0 (0%)	0 (0%)	
incomplete structural	19 (52.8%)	7 (46.7%)	12 (57.1%)	
indeterminate	3 (8.3%)	3 (20.0%)	0 (0%)	
PTC-related death	12 (33.3%)	6 (40.0%)	6 (28.6%)	0.499

PTC—papillary thyroid cancer; CNS—central nervous system; RAI—radioiodine; ATA – American Thyroid Association. * ATA treatment response was evaluated accordance to “2015 ATA Management Guidelines for Adult Patients with Thyroid Nodules and Differentiated Thyroid Cancer” [7].

## Supplementary Materials

Supplementary materials can be found at https://www.mdpi.com/1422-0067/21/13/4629/s1.

## Abbreviations

PTC	Papillary thyroid cancer
M1	Distant metastases present
M0	Distant metastases absent
qPCR	Quantitative polymerase chain reaction
DTC	Differentiated thyroid cancer
TNM	Tumor, nodes, metastasis
ATA	American thyroid association
HG-U133A	Affymetrix human genome U133A arrays
HG-U133 Plus 2.0	Affymetrix human genome U-133 plus 2.0 arrays
SLR	Signal log ratios
FDR	False discovery rate
FC	Fold change
lnc-RNA	Long non-coding RNA
RNA-Seq	RNA sequencing
CTC	Circulating tumor cells
CNS	Central nervous system
RAI	Radioiodine
KEGG	The Kyoto Encyclopedia of Genes and Genomes
GSEA	Gene Set Enrichment Analysis
NES	Normalized Enrichment Score
