# Supplementary material for: Differences in Gene Expression Profile of Primary Tumors in Metastatic and Non-Metastatic Papillary Thyroid Carcinoma—Do They Exist?
Source: Int J Mol Sci. 2020 Jun 29;21(13):4629. doi: 10.3390/ijms21134629 (PMC7369779; doi:10.3390/ijms21134629)
Supplement: Supplementary file 1 [file ijms-21-04629-s001.pdf]

Table S1. List of 28 genes validated by qPCR. No significant gene was found (data not shown).

Gene	Full gene name
ACACA	acetyl-Coenzyme A carboxylase alpha
AMACR	alpha-methylacyl-CoA racemase
ARHGEF2	rho/rac guanine nucleotide exchange factor (GEF) 2
BLVRA	biliverdin reductase A
CASK	calcium/calmodulin-dependent serine protein kinase (MAGUK family)
CCT5	chaperonin containing TCP1, subunit 5 (epsilon)
CDC2	cell division cycle 2, G1 to S and G2 to M
CIB1	calcium and integrin binding 1 (calmyrin)
CRELD2	homo sapiens cysteine rich with EGF like domains 2
GALNT10	UDP-N-acetyl-alpha-D-galactosamine:polypeptide N-acetylgalactosaminyltransferase 10
GUSB	glucuronidase, beta
HMMR	hyaluronan-mediated motility receptor
IGFBP3	insulin-like growth factor binding protein 3
ITPR3	inositol 1,4,5-triphosphate receptor, type 3
KLK10	kallikrein-related peptidase 10
KPNA6	karyopherin alpha 6 (importin alpha 7)
LAMP1	lysosomal-associated membrane protein 1
P4HB	procollagen-proline, 2-oxoglutarate 4-dioxygenase (proline 4-hydroxylase), beta polypeptide
PARP8	poly (ADP-ribose) polymerase family, member 8
PLCH1	phospholipase C, eta 1
RPN2	ribophorin II
SERTAD2	SERTA domain containing 2
SLC2A8	solute carrier family 2, (facilitated glucose transporter) member 8
SMOX	spermine oxidase
SNCA	synuclein, alpha (non A4 component of amyloid precursor)
SRI	Sorcin
TRPS1	trichorhinophalangeal syndrome I
TXNRD3	thioredoxin reductase 3

Table S2. Clinical characteristic of the qPCR group. .

	All QPCR patients	M1 patients	M0 patients	*p*-value
Patients number	63	9	54	
Median age at diagnosis [years] (range)	50 (12-75)	29 (12-66)	53 (12-75)	0.091
Median follow-up [months] (range)	148.3 (18.0-238.4)	135.1 (40.6-238.4)	148.9 (18.0-184.0)	0.875
Sex:				
- male	14 (22.2%)	5 (55.6%)	9 (16.7%)	0.020
- female	49 (77.8%)	4 (44.4%)	45 (83.3%)	
PTC histological subtype				
- classic	51 (81.0%)	6 (66.7%)	45 (83.3%)	
- follicular	9 (14.3%)	3 (33.3%)	6 (11.1%)	0.158
- other	3 (4.8%)	0 (0%)	3 (5.6%)	
T feature (primary tumor):				
- T1	39 (61.9%)	1 (11.1%)	38 (70.4%)	
- T2	11 (17.5%)	2 (22.2%)	9 (16.7%)	
- T3	8 (12.7%)	2 (22.2%)	6 (11.1%)	< 0.001
- T4	3 (4.8%)	2 (22.2%)	1 (1.9%)	
- Tx	2 (3.2%)	2 (22.2%)	0 (0%)	
Lymph node metastases:				
- central neck compartment and upper mediastinum (N1a)	24 (38.1%)	7 (77.8%)	17 (31.5%)	0.003
- lateral neck compartment and retropharyngeal lymph nodes (N1b)	21 (33.3%)	7 (77.8%)	14 (25.9%)	0.001
Recurrence after primary treatment	6 (9.5%)	1 (11.1%)	5 (9.3%)	> 0.999
PTC-related death	3 (4.8%)	3 (33.3%)	0 (0%)	0.002

The patients were staged according to the 8th UICC/AJCC TNM Edition (2016). PTC – papillary thyroid cancer; M1 – distant metastases present; M0 - distant metastases absent

Table S3. List of primers used in qPCR.

Gene	Primers sequence (5′-3′)		Lenght (bp)
ACACA	F	gatgtggatgatgggctaca	63
	R	tgaggccttgatcattactgg	
AMACR	F	tagtgctggacctgaagcag	112
	R	agctggagtttctccatgaca	
ARHGEF2	F	gtgctatgcctgtaacaagagc	72
	R	tggatagtcacattgcaggttg	
BLVRA	F	aggtcgcctatatctgcagtg	71
	R	cttgccagcattaaggaactg	
CASK	F	caattaggggagtctggacttg	63
	R	tggtgccataaaatgaggtg	
CCT5	F	attggagatggaaccacagg	66
	R	caattgctccgcttcttctaa	
CDC2	F	tggatctgaagaaatacttggattcta	96
	R	caatcccctgtaggatttgg	
CIB1	F	acaggcggttttgtgagc	123
	R	gctccttgaaggggttgg	
CRELD2	F	gcttcgaaggaacggaagat	72
	R	gtgtcgggctttctccttct	
GALNT10	F	ggaagtatgtgccctacaaggt	64
	R	gccacccgcttaaggttc	
GUSB	F	cgccctgcctatctgtattc	91
	R	tccccacagggagtgtgtag	
HMMR	F	cgattcaatgacccttctgg	85
	R	aggatactggtcctttcaatacttct	
IGFBP3	F	aacgctagtgccgtcagc	86
	R	cggtcttcctccgactcac	
ITPR3	F	tgtgctggacaccatcaac	94
	R	cagcagctgcaccacaac	
KLK10	F	ttccctaccgctgtgctc	95
	R	ggtcaggcccttgttgtact	
KPNA6	F	tcagactttgaggatgttcagg	73
	R	ggcaaacagagctatctccag	
LAMP1	F	gtgggtccaggctttcaa	78
	R	agcatgctgttctcgtcca	
P4HB	F	gcttcccccaaggaatataca	61
	R	tcttcagccagttcacgatg	
PARP8	F	agggtttgaaaagcaaacaca	71

Gene		Primers sequence (5′-3′)	Lenght (bp)
	R	agggtttgaaaagcaaacaca	
PLCH1	F	aactgtggaagaactggctca	74
	R	caagacaatagtccgttgtcacat	
RPN2	F	ggcagtgtgacgtttctgg	76
	R	tctggaactaatgtgctgttctct	
SERTAD2	F	ccctcctgatgcgttagttc	66
	R	cgttttcctcctttacccaac	
SLC2A8	F	ggcctttctcgtgaccaa	71
	R	caagccagaaggctccatag	
SMOX	F	gatgctgcgtcagttcacag	95
	R	aggagccgcggaagtaag	
SNCA	F	caaacagggtgtggcaga	72
	R	tccttggttttggagcctac	
SRI	F	aaagatcaccttcgacgactaca	86
	R	gctgagcagtatcccgtctt	
TRPS1	F	cgtgtggcctctaccagaag	109
	R	gggttaaggcgctttcttg	
TXNRD3	F	aaggaaatttggctgggaat	74
	R	ggttctgaatcgcttttgtca	
